# Alpha-Synuclein RNA Expression is Increased in Major Depression

**DOI:** 10.3390/ijms20082029

**Published:** 2019-04-25

**Authors:** Andrea Rotter, Bernd Lenz, Ruben Pitsch, Tanja Richter-Schmidinger, Johannes Kornhuber, Cosima Rhein

**Affiliations:** Department of Psychiatry and Psychotherapy, Friedrich-Alexander-University Erlangen-Nürnberg (FAU), D-91054 Erlangen, Germany; andrea.rotter-neubert@Landratsamt-Roth.de (A.R.); bernd.lenz@uk-erlangen.de (B.L.); rubenpitsch@gmail.com (R.P.); tanja.richter-schmidinger@uk-erlangen.de (T.R.-S.); johannes.kornhuber@uk-erlangen.de (J.K.)

**Keywords:** alpha-synuclein, SNCA, major depression, Hamilton Scale of Depression

## Abstract

Alpha-synuclein (SNCA) is a small membrane protein that plays an important role in neuro-psychiatric diseases. It is best known for its abnormal subcellular aggregation in Lewy bodies that serves as a hallmark of Parkinson’s disease (PD). Due to the high comorbidity of PD with depression, we investigated the role of SNCA in patients suffering from major depressive disorder (MDD). *SNCA* mRNA expression levels were analyzed in peripheral blood cells of MDD patients and a healthy control group. *SNCA* mRNA expression was positively correlated with severity of depression as indicated by psychometric assessment. We found a significant increase in *SNCA* mRNA expression levels in severely depressed patients compared with controls. Thus, SNCA analysis could be a helpful target in the search for biomarkers of MDD.

## 1. Introduction

Alpha-synuclein (SNCA) is a small membrane protein (~14 kDa) consisting of 140 amino acids encoded on chromosome 4q21 [1,2,3,4]. It was shown that SNCA is localized close to synaptic vesicles and interacts with the cell membrane. A specific role in the regulation of dopamine transmission was suggested [5]. SNCA was further found to localize at neuronal growth cones, which indicates a role in neuronal plasticity [6,7,8,9,10]. Abnormal subcellular SNCA aggregation is a hallmark of neurodegenerative diseases (Parkinson’s disease, dementia with Lewy bodies, and multiple system atrophy) that are recognized as alpha-synucleinopathies [11]. In addition, there is increasing evidence that SNCA could also be involved in the pathophysiology of major depressive disorder (MDD). MDD is a severe psychiatric disorder with a lifetime prevalence of approximately 10% that is characterized by depressed mood, a decline in motivation and the loss of feelings of pleasure and interest, resulting in increased suicide rates [12]. Due to the unclear pathogenesis of MDD it is suggested that environmental factors such as psychosocial stress and genetic characteristics trigger dysregulation of the cytokine system, the neurotransmitter systems, the hormonal systems and the circadian rhythm [13,14,15,16]. It has been shown that in all alpha-synucleinopathies, there is a 30–60% comorbidity with MDD [11]. An involvement of SNCA in psychiatric disorders was first detected in a study about eating disorders that correlated *SNCA* mRNA levels positively with the severity of depressive symptoms [17]. The connecting link between SNCA and MDD could be its modulating effect on monoamine transporters [18]. SNCA influences the expression and, thereby, the activity of dopamine, serotonin and norepinephrine transporters through direct binding and influence on trafficking, and helps to maintain the homeostasis of monoamine neurotransmitters in the brain [19,20,21]. SNCA was also associated with stress in a rat model of depression [22]. In addition, antidepressant therapy influences the SNCA system. Desipramine has been shown to modulate SNCA and the norepinephrine transporter in an animal model of depression [23]. Antidepressant therapy was found to influence *SNCA* mRNA expression in the hippocampus of rats [24]. Rats that were treated with paroxetine showed decreased protein expression of SNCA [25]. Several *SNCA* single nucleotide polymorphisms (SNPs) have been identified and seem to play an important role in the pathophysiology of psychiatric diseases. Alcohol craving, for example, was shown to be associated with an SNP in the *SNCA* gene [4] and higher SNCA protein levels in patients [26]. Moreover, significantly longer alleles of the repeat NACP-REP1 were detected in alcohol-dependent patients compared with healthy controls [27]. The NACP-REP1 length polymorphism was also found to correlate with depressive symptoms in healthy volunteers [15]. GWAS studies have identified *SNCA* as one of the top genes relevant to psychiatric disorders [28]. 

Therefore, we hypothesized a role for SNCA in MDD and investigated the link between *SNCA* mRNA expression in peripheral blood and depressive symptoms in depressed patients. 

## 2. Results

### 2.1. SNCA mRNA Expression Correlates Positively with the Severity of Depressive Symptoms

The mRNA expression levels of *SNCA* were determined in peripheral blood cells of MDD patients. The severity of depressive symptoms in patients was assessed using two psychometric scales: the Hamilton depression rating scale (HAM-D-17) for clinician-administered rating and Beck’s Depression Inventory—revised (BDI-II) for self-report rating. To investigate the relationship between *SNCA* mRNA expression and the severity of depressive symptoms in patients, we conducted a correlative analysis. Using Pearson correlation, we found a significant correlation between *SNCA* mRNA expression levels and BDI-II (*r* = 0.281, *p* = 0.026) and HAM-D-17 scores in the patients (*r* = 0.273, *p* = 0.028). Thus, the severity of depressive symptoms in MDD patients, as indicated by a higher psychometric score in the self-report rating as well as in the clinician-administered rating, was positively correlated with the measured *SNCA* mRNA expression in their blood cells.

### 2.2. SNCA mRNA Expression is Increased in Patients with Severe Depression

When analyzing the data of the MDD patients more closely, it turned out that the two patient subgroups, “ADT” (MDD patients recruited for the study “AntiDepressive Therapy” (ADT)) and “BLADe” (MDD patients participating in the study “Blood Lipid Alterations in Depression” (BLADe), Table 1) differed not only with regard to treatment, but also with regard to severity of symptoms. The patients of the ADT study, who were untreated, had an average HAM-D score of 17.7 ± 8.2 points, which characterizes this group as being moderately depressed. In contrast, the patients of the BLADe study, who were already treated at the beginning of the study, had an average HAM-D score of 21.4 ± 5.2 points, which falls into the category of severe depression. The difference in the severity of symptoms between both groups was statistically significant, as the ADT group exhibited significantly lower HAM-D scores than the BLADe group (*t*-test, df = 63, T = 2.2, *p* = 0.031; Table 2). There was no significant difference between females and males regarding the severity of depression (HAM-D score of 21.3 ± 6.6 and 18.0 ± 6.7, respectively; *t*-test, df = 63, T = −1.9, *p* > 0.05).

In a further analysis, we assessed the difference in *SNCA* mRNA expression between patients and controls. Due to the significant difference in age between the BLADe patient group and the healthy volunteers (Table 1), age was included as a covariate in all analyses. *SNCA* mRNA expression values were normally distributed. Compared with the control group, which had a mean normalized *SNCA* mRNA expression level of 17.4 ± 5.4 in their blood cells, MDD patients in the BLADe and ADT studies displayed increased *SNCA* mRNA expression levels (mean normalized expression of 31.9 ± 15.3 and 24.3 ± 13.8, respectively; analysis of variance (ANOVA) df = 2, F = 5.9, *p* = 0.004; Table 2). Pairwise comparison analysis revealed that the significant difference resulted from the comparison of the control group with the BLADe patient group (*p* = 0.001), but not with the ADT patient group (*p* = 0.114). Moreover, the comparison of *SNCA* mRNA expression between both patient groups revealed that the BLADe subgroup showed significantly higher *SNCA* mRNA expression levels than the ADT subgroup (*p* = 0.031). Even though females had a higher level of *SNCA* mRNA expression compared with males (mean normalized expression of 31.0 ± 17.2 and 21.3 ± 8.1, respectively; *t*-test, df = 63, T = −3.4, *p* = 0.001), there was no interaction effect between the groups and sex. Therefore, *SNCA* mRNA expression differs between healthy controls and depressed patients and seems to increase with the severity of depressive symptoms. 

## 3. Discussion

Our study shows for the first time a significant increase in *SNCA* mRNA expression levels in severely depressed patients compared with healthy controls. This is in line with a parallel study in which increased SNCA protein levels were measured in the blood serum of depressed patients [29]. Our results showing a positive correlation between *SNCA* mRNA expression and BDI-II scores confirm a study by Frieling and colleagues in which this relationship was found in eating disorders [17]. Of note, other data have shown that patients who exhibited an early remission upon antidepressant treatment had increased *SNCA* mRNA expression levels at baseline compared with a non-responder group, but *SNCA* mRNA expression was not monitored during and after treatment [30]. The insights from clinical studies are derived from analyses of peripheral blood cells and do not address central mechanisms. In a murine study, the overexpression of SNCA in midbrain dopaminergic neurons resulted in depressive-like behavior [31]. The mediating effect of increased SNCA on the development of depression may be related to impaired adult neurogenesis in the hippocampus. In a mouse model overexpressing mutant A53T SNCA, adult neurogenesis in the dentate gyrus of the hippocampus was significantly impaired due to a reduction in proliferation of neural stem and precursor cells [32]. Another link could involve compromised neurotransmitter release associated with increased SNCA. In a stress model of depression in rats, several proteins were found to be differentially expressed and associated with deficits in synaptic vesicle release involving SNCA, synapsin I and the adaptor protein-3 complex, which were hypothesized to contribute to the pathomechanisms of psychiatric diseases [22]. 

Interestingly, increased mRNA expression of *SNCA* in patients were also detected in studies focusing on other neuro-psychiatric diseases: in neuronal disorders [3], in alcohol dependence [4] and cocaine dependence [33]. A common hallmark of these diseases is the impairment of cognition. The high comorbidity of PD with dementia and depression thus points to a common pathway. In 30–60% of PD patients, depressive symptoms occur and often precede motor symptoms [34]. The lack of studies investigating PD patients with depression makes further insights difficult. The treatment of depression in PD was investigated in two studies that found better outcomes for tricyclic antidepressants than for selective serotonin reuptake inhibitors (SSRIs) [35,36]. In murine studies, it was shown that treatment with fluoxetine did not influence *SNCA* mRNA expression levels [32], whereas paroxetine decreased *SNCA* mRNA levels [25]. It could be hypothesized that antidepressants in PD work via an influence on SNCA levels and that fluoxetine and paroxetine classified as SSRI are not optimized for this effect. Additionally, in a mouse model overexpressing SNCA, serotonergic projections in the hippocampus seem to be compromised, and the high protein levels of SNCA affected responsiveness to SSRIs [37]. These conflicting results indicate the need for human studies that monitor SNCA levels after antidepressant treatment. 

One limitation of the present study may be that the number of patients in this study was relatively small, and we did not monitor treatment effects on *SNCA* mRNA expression. Moreover, the healthy volunteers differed from one of the patient groups regarding age. 

In summary, we show a significant increase in *SNCA* mRNA expression levels in patients suffering from severe depression. Further studies with larger sample sizes and treatment monitoring are warranted to elucidate the clinical relevance of SNCA in MDD.

## 4. Materials and Methods

### 4.1. Ethics Statement

The collection of blood samples was approved by the Ethics Committee of the Friedrich-Alexander-University Erlangen-Nürnberg (FAU) (ID 4194, renewal of 3412, approval date: 20 April 2010) and conducted in concordance with the Declaration of Helsinki. Written informed consent was obtained from all participants.

### 4.2. Study Sample

All patients had an established diagnosis of MDD according to the International Statistical Classification of Diseases and Related Health Problems (ICD-10) and the Diagnostic and Statistical Manual of Mental Disorders (DSM-IV) criteria. After hospital admission, diagnosis was confirmed by conducting a diagnostic interview using the *Strukturiertes Klinisches Interview für DSM-IV* (SKID-I). Further, the following clinical scales were administered: the Hamilton depression rating scale (HAM-D-17) for clinician-administered rating and Beck’s Depression Inventory—revised (BDI-II) for self-report rating. All participants were carefully screened to rule out the existence of inflammatory, cardiac, endocrine, renal and hepatic disease by means of a structured medical history, physical examination, routine laboratory testing, and electrocardiography. Patients were excluded if comorbidity of alcohol or drug dependence was detected. MDD patients participating in the BLADe (Blood Lipid Alterations in DEpression) study (*n* = 39) were already treated with a standard antidepressant therapy at admission. MDD patients recruited for the ADT (AntiDepressive Therapy) study (*n* = 31) had not been treated with antidepressants, and standard antidepressant therapy was initiated after taking blood samples. A group of 18 healthy subjects without a personal history of psychiatric and somatic disorders served as a control group [38]. The BLADe patient group differed significantly from the healthy volunteers in terms of age (ANOVA, df = 2, F = 7.8, *p* = 0.001; post hoc analysis revealed significant difference only between controls and the BLADe group, *p* = 0.001; Table 1).

### 4.3. RNA Isolation and cDNA Synthesis

For patients in the ADT study, blood of fasting patients was taken for RNA isolation in the morning to secure for stable experimental conditions. For RNA isolation, the PAXgene system was employed (PreAnalytiX GmbH, Hombrechikon, Switzerland). PAXgene tubes containing blood samples were incubated at room temperature for 2 h, stored at −80 °C, and RNA was isolated according to manufacturer’s instructions. For patients in the BLADe study and for control samples, total RNA was extracted from whole blood in EDTA using Qiacube and the accordant protocol (QIAGEN GmbH, Hilden, Germany). RNA quality and quantity were analyzed using the Experion TM Automated Electrophoresis System and Nanodrop 1000 (PEQLAB, Erlangen, Germany). Reverse transcription was performed using the Bio-Rad Laboratories’ iScript cDNA Synthesis Kit (Bio-Rad, Munich, Germany).

### 4.4. Quantitative PCR

The expression of *SNCA* was analyzed by quantitative PCR using the LightCycler System (LightCycler^®^ SW 1.5, Roche Diagnostics GmbH, Mannheim, Germany) as previously described [39]. Briefly, *SNCA* expression was assessed using SYBR green technology (Bio-Rad, Munich, Germany), and the mean of beta-actin (*B-Actin*), beta-2-microglobulin (*B2M*) and ornithine decarboxylase 1 (*ODC1*) expression values, assessed using specific probes of the Roche Universal Probe Library (Roche Diagnostics GmbH, Mannheim, Germany), served as reference values (Table 3). Mean normalized expression was calculated using the “Abs Quant/2nd Derivative Max” analysis method provided by Roche (Mannheim, Germany). 

### 4.5. Statistical Analysis

Variables were tested for deviation from the normal distribution using the Kolmogorov-Smirnov test. Differences in sex distribution were calculated using the chi quadrat test. Correlative analyses were conducted using Pearson correlation coefficient. T-test and analysis of variance (ANOVA) were used to test for differences between the groups. A two-sided *p*-value ≤ 0.05 was considered indicative of statistical significance. The data were analyzed using SPSS TM for Windows 18.0 (SPSS Inc., Chicago, Ill., USA).

## Figures and Tables

**Table 1:** Demographic overview. Differences in sex distribution were calculated using the chi quadrat test. Differences regarding age were calculated using analysis of variance (ANOVA). SD, standard deviation. ADT, MDD patients recruited for the study “AntiDepressive Therapy”; BLADe, MDD patients participating in the study “Blood Lipid Alterations in Depression”.

	BLADe	ADT	Healthy Controls	*p*-Value
N (male/female)	39 (15/24)	31 (15/16)	18 (13/5)	0.060
Age (years ± SD)	46.3 ± 14.2	39.7 ± 16.5	30.4 ± 8.8	0.001

**Table 2:** Values for *SNCA* mRNA expression and psychometric scores. *SNCA* mRNA expression and Hamilton depression rating scale (HAM-D) scores differ significantly between groups (ANOVA). HAM-D was not conducted in the control group.

	BLADe	ADT	Healthy Controls	*p*-Value
*SNCA* expression ± SD	31.9 ± 15.3	24.3 ± 13.8	17.4 ± 5.4	0.004
HAM-D scores ± SD	21.4 ± 5.2	17.9 ± 8.2	-	0.034

**Table 3:** Sequences of oligonucleotides employed.

**SNCA-F**	5′-CTC CTT TTC CTT CTT CTT TCC T-3′
**SNCA-R**	5′-TGT TTG GTT TTC TCA GCA GC-3′
**B-Actin-F**	5′-GTC TTC CCC TCC ATC GTG-3′
**B-Actin-R**	5′-AGG TGT GGT GCC AGA TTT TC-3′
**B-Actin-probe**	5′ Cy5-GAG CAA GAG AGG CAT CCT CAC CCT GAA GTA-Eclipse 3′
**ODC1-F**	5′-CGC TTA CAC TGT TGC TGC TG-3′
**ODC1-R**	5′-CAT CCT GTT CCT CTA CTT CGG G-3′
**ODC1-probe**	5′ HEX-TCC AGA GGC CGA CGA TCT ACT ATG TGA TGT-BHQ1 3′
**B2M-F**	5′-CGC TAC TCTC TCT TTC TGG C-3′
**B2M-R**	5′-GTC AAC TTC AAT GTC GGA TGG AT-3′
**B2M-probe**	#42 of Roche Universal Probe Library
